# Repeated electromagnetic field stimulation lowers amyloid-β peptide levels in primary human mixed brain tissue cultures

**DOI:** 10.1038/s41598-020-77808-2

**Published:** 2021-01-12

**Authors:** Felipe P. Perez, Bryan Maloney, Nipun Chopra, Jorge J. Morisaki, Debomoy K. Lahiri

**Affiliations:** 1 Indiana University School of Medicine, Indianapolis, IN USA; 2 Department of Medicine, Division of General Internal Medicine and Geriatrics, Indiana University School of Medicine, Indianapolis, IN USA; 3 Department of Psychiatry, Institute of Psychiatric Research, Neuroscience Research Center, Indiana University School of Medicine, 320 W. 15th St, Indianapolis, IN 46201 USA; 4 Department of Bioengineering, University of Illinois at Chicago, Chicago, IL USA; 5 Department of Medical and Molecular Genetics, Indiana University School of Medicine, Indianapolis, IN USA

**Keywords:** Neurological disorders, Psychiatric disorders, Neuroscience, Alzheimer's disease

## Abstract

Late Onset Alzheimer’s Disease is the most common cause of dementia, characterized by extracellular deposition of plaques primarily of amyloid-β (Aβ) peptide and tangles primarily of hyperphosphorylated tau protein. We present data to suggest a noninvasive strategy to decrease potentially toxic Aβ levels, using repeated electromagnetic field stimulation (REMFS) in primary human brain (PHB) cultures. We examined effects of REMFS on Aβ levels (Aβ40 and Aβ42, that are 40 or 42 amino acid residues in length, respectively) in PHB cultures at different frequencies, powers, and specific absorption rates (SAR). PHB cultures at day in vitro 7 (DIV7) treated with 64 MHz, and 1 hour daily for 14 days (DIV 21) had significantly reduced levels of secreted Aβ40 (*p* = 001) and Aβ42 (*p* = 0.029) peptides, compared to untreated cultures. PHB cultures (DIV7) treated at 64 MHz, for 1 or 2 hour during 14 days also produced significantly lower Aβ levels. PHB cultures (DIV28) treated with 64 MHz 1 hour/day during 4 or 8 days produced a similar significant reduction in Aβ40 levels. 0.4 W/kg was the minimum SAR required to produce a biological effect. Exposure did not result in cellular toxicity nor significant changes in secreted Aβ precursor protein-α (sAPPα) levels, suggesting the decrease in Aβ did not likely result from redirection toward the α-secretase pathway. EMF frequency and power used in our work is utilized in human magnetic resonance imaging (MRI, thus suggesting REMFS can be further developed in clinical settings to modulate Aβ deposition.

## Introduction

In the United States alone, there are over 5.8 million individuals with AD, and numbers are expected to rise in parallel with life expectancy^1^. The number of people living with Alzheimer’s disease (AD) and other dementias worldwide was estimated at 46 million in 2015, with estimated prevalence reaching 131 million in 2050^2^. The total estimated worldwide cost of dementia was $604 billion in 2010. Barring development of medical breakthroughs to prevent, slow down, or stop the disease, potential impacts on health, society, and the global economy will be enormous.

AD is a complex and heterogeneous disorder that includes both familial autosomal dominant early-onset (EOAD), and sporadic late-onset AD (LOAD); the latter being far more common^2^. Although age is the most closely associated factor, the specific etiology of LOAD, distinct from overall aging is presently unknown, several factors, including genetic, epigenetic, lifestyle and environment, are thought to be associated with AD^3,4^. AD is characterized by neuritic plaques of amyloid-β (Aβ) peptide, neurofibrillary tangles of hyperphosphorylated microtubule-associated protein τ, gliosis, neuroinflammation, and synaptic loss^5–7^. Aβ is cleaved sequentially from the Aβ precursor protein (APP) by β-secretase (BACE1) and γ-secretase complex. This “amyloidogenic” processing pathway is neurodegenerative. In contrast, the “anabolic” pathway wherein APP is processed first by one of the α-secretases, followed by γ-secretase activity, is neuroprotective and neurotrophic^8–12^.

Currently available treatments for AD have demonstrated limited efficacy. The drugs approved by the U.S. Food and Drug Administration (FDA) for the treatment of some symptoms of AD at best only improve them temporarily^13^, and their effectiveness varies across patients. Although none of the available treatments significantly alter progression of the disease, lessons from recent failed clinical drug trials have provided important clues and prompted researchers to reexamine some of these strategies, such as antibody treatment against Aβ or inhibition of BACE1^14,15^. In addition to pharmacological approaches, researchers are examining alternative modalities to slow or halt the disease process. These include “holistic” approaches and lifestyle modifications that seek to improve diet, exercise, and social enrichment^3^.

Other non-pharmacological interventions for management of neuropsychiatric disorders, including AD, major depressive disorder and autism spectrum disorder^16,17^ include transcranial magnetic stimulation (TMS). TMS is approved for the treatment of treatment resistant depression. Given its tolerability, there is a growing interest to explore other potential applications and their mechanisms. Several well written reviews summarize TMS and other stimulation modalities^18^.

Recently a 2-month phase 1 clinical trial of electromagnetic exposure (915 MHz) to AD patients for 1 hour (h) twice a day found no deleterious behavioral effects, discomfort, or physiologic changes^19^. Such noninvasive, non-pharmacological approaches are inherently appealing if they were to improve cognition in AD. Active research in the use of noninvasive brain stimulation as a potential therapy for AD has included a number of pilot studies and small clinical trials that have highlighted the potential for neuroenhancement and improvement in cognitive function in healthy individuals via noninvasive brain stimulation (NBS)^20^. However, consensus is lacking on their mechanism of action, efficacy and reproducibility^21–24^.

Our goal is to explore the use of NBS, such as repeated electromagnetic field stimulation (REFMS), as a potential non-invasive strategy to lower Aβ peptide load observed in AD. The present work aims at studying the neurobiological effects of REFMS on neuronal cell viability, and its effect on levels of potentially toxic Aβ peptide in primary human brain cultures. Recent AD animal experiments suggest that REMFS could potentially be a disease modifying and safe strategy^25–29^. In mouse models REMFS at a frequency of 918 MHz^27–29^ and 1950 MHz^25,26^ protected against and reversed cognitive impairment by decreasing Aβ amyloid deposition. Also, other investigators have found that REMFS exposure attenuates tau phosphorylation in the hippocampus of AD mice^30^, thus suggesting beneficial in vivo effects of REMFS in age-related AD-like mouse pathology.

The influence of REMFS on biological systems entails thermal and non-thermal effects. Its thermal effect depends primarily on the specific absorption rates (SAR). Whereas its non-thermal biological effect occurs at the molecular level, and involves multitarget interactions between signaling pathways^31–34^, including those between EMF-DNA^35^, EMF-RNA^36^, in addition to changes in Ca^2^^+^ regulation^37,38^, channel activity^39^, enzyme activity^40^, nucleic acid synthesis^41–43^, and microRNA expression^44–46^. Other changes include free radical gene expression^47–49^, oxidative stress reduction^50–54^, heat shock response^55^, heat shock factor 1 activation^56^, and mTOR activation^57^. Likewise, other molecular effects are noteworthy, such as histone acetylation^58^, cell protection^59^, growth behavior^60,61^, ubiquitin–proteasome system activation^62–64^, autophagy-lysosome systems^36^, inflammation^65–67^, mitochondrial enhancement^27^, neuronal activity^28^. Particularly, in the context of our studies on the APP pathway, BACE1 mRNA reduction^44^, regulation of gene expression^68^, and epigenetic alterations^69,70^, are important.

Many different types of cells that respond to EMF exposures^71,72^, the present study adds primary human neurons and glia to the growing list, and attempts to establish the lowest SAR capable of producing potentially specific, non-thermal effects. As discussed in our mathematical model and computer simulation articles, we calculated the applied SAR to our cell cultures^34,73^, a 64 MHz frequency allowed us to minimize power needed to obtain the minimum SAR with biological effect, often called “MSBE”, permitting the use of an average SAR that was well below the permitted values of 2 W/kg (Watts per kilogram) set forth by the International Electrotechnical Commission (IEC). Thus, our conditions provide an established framework for safe human exposure (Table 1).

**Table 1:** Electromagnetic frequencies and human tissue penetration^77^.

Frequency	Depth of Penetration (cm) into various tissues												
	Skin (Dry)	Fat	Muscle	Skeletal System			Nervous System						
				(Bone)			CNS						Peripheral
				Cancellous	Cortical	Marrow	Dura	Cerebellum	Gray Matter	White Matter	Spinal Cord	CSF	Nerve
64 MHz	13.5	45.6	9.1	21.6	40.0	72.4	9.0	9.8	12.1	17.0	15.1	4.8	15.1
100 MHz	10.5	40.0	7.7	18.1	34.3	62.1	7.3	7.6	9.7	13.6	12.4	3.9	12.4
918 MHz	4.0	24.2	4.2	7.1	13.0	30.7	3.7	3.0	4.1	5.6	5.3	1.9	5.3

When applied experimentally within these parameters, REMFS resulted in significant reductions in levels of both Aβ40 and Aβ42 peptides, with possible little perturbation of cell culture vitality and health. Interestingly, no alterations in total processed APP levels, as measured by secreted APP-α (sAPPα) or total secreted APP (sAPP), were observed. A mechanism for this biophysical interaction currently remains unknown, but may involve increased Aβ degradation due to activation of several proteolytic pathways^34,36,62–64,74,75^. Alternatively, a complex readjustment of secretase activity may also play a role. EMF frequency and power used in our work is typical of that already utilized in human magnetic resonance imaging (MRI, thus suggesting REMFS can be further developed in appropriate animal models and clinical settings to modulate Aβ deposition. Our work is, thus, both mechanistic and translational, and would advance the field of neuroscience as well as AD.

## Results

### REMFS treatment was not toxic in primary human brain cells

**Figure 1:**
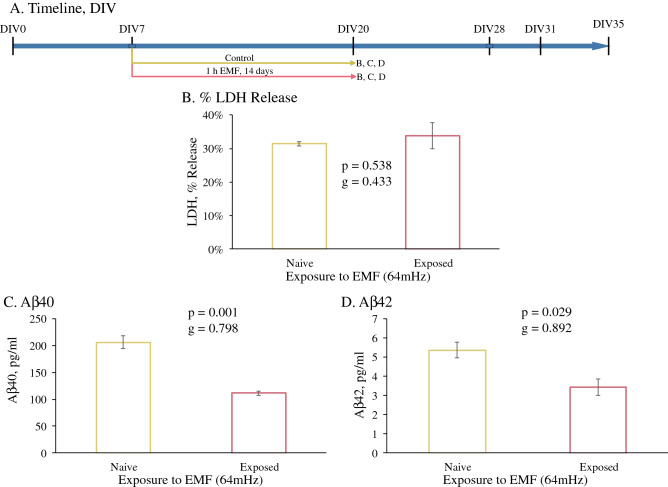
REMFS effects on cellular toxicity and Aβ40 and 42 levels. (**A**) Schedule of cell growth and treatments for data presented in figure. (**B**) REMFS treatment at 64 MHz with a SAR of 0.6 W/kg daily for 1 h for 14 days did not show significant cellular toxicity and/or membrane damage by LDH assay. (**C**) REMFS at 64 MHz with a SAR 0.6 W/kg daily for 1 h for 14 days reduced Aβ40 levels in PHB tissue culture conditioned media (*p* = 0.001). (**D**) REMFS reduced Aβ42 levels in PHB cell culture conditioned media (*p* = 0.029).

PHB cultures were utilized to investigate the effects on levels of potentially toxic secreted Aβ peptide. An example of PHB culture morphology and cell type distribution has been published^76^. PHB cultures were subjected to REMFS at 64 MHz with a SAR of 0.6 W/kg every day for 1 h in the TEM (Transversal Electromagnetic) cell chamber, which was performed initially in an incubator and after determining no significant difference, at room temperature (Fig. 1A). Cell membrane damage and integrity were measured by assaying lactate dehydrogenase (LDH) release into the conditioned medium (CM) in comparison to LDH present in cell lysates collected at the end of the experiment. We observed no significant difference in relative %LDH released between non-treated and REMFS-treated samples at DIV21 (14 days EMF treatment) (Fig. 1B).

### REMFS lowered Aβ40 and Aβ42 levels in PHB cultures

We measured levels of Aβ40 peptide in CM samples via ELISA after 14 day of exposure in treated and non- treated cultures, beginning at DIV 7 (Fig. 1A). The REMFS dose tested in this study, 64 MHz with a SAR of 0.6 W/kg, 1 h daily over a 14-day period, yielded a 46% decrease in Aβ40 levels in the three independent experiments examined (Fig. 1C, *p*  = 0.001, *g* = 0.798), compared to the non-treated cultures. The same treatment produced a 36% reduction in Aβ42 levels (Fig. 1D,* p* = 0.029, *g* = 0.892).

### Daily REMFS for 14 days at different lengths of exposure was non-toxic to cells and reduced Aβ40 and Aβ42 levels

**Figure 2:**
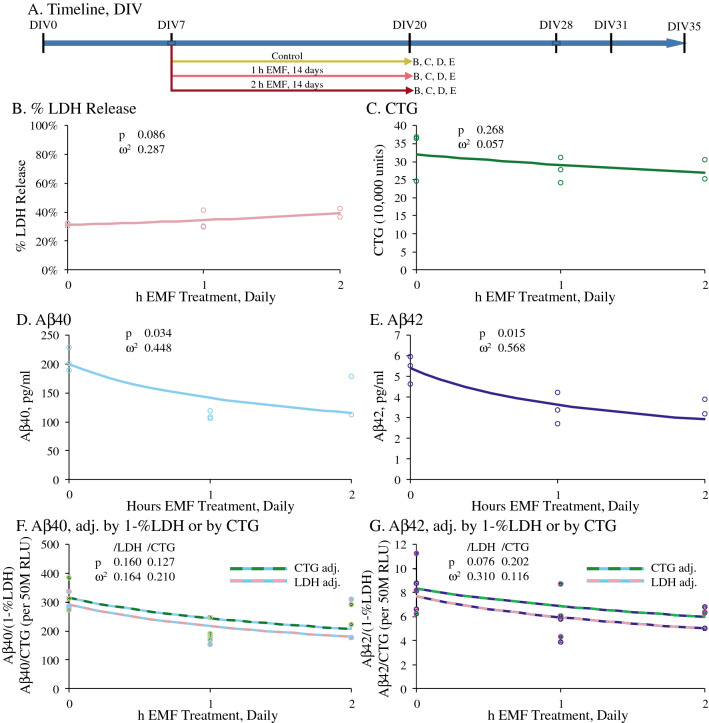
REMFS effects on Aβ40 and Aβ versus different exposure times in human brain cultures. PHB cultures were treated for 1 or 2 h at 64 MHz with a SAR of 0.4 W/kg and secreted Aβ40 and Aβ42 were measured in CM by ELISA as described in the text. Data is presented as individual measurements and corresponding regression lines. (**A**) Schedule of cell growth and treatments for data presented in figure. (**B**) LDH assay showed that REMFS treatment did not cause cell toxicity. (**C**) CTG data showed no significant change in cell viability dependent on exposure time. (**D**) Aβ40 versus daily exposure time. Aβ40 significantly (*p* = 0.034) decreased as EMF exposure time increased. (**E**) Aβ42 versus daily exposure time. Aβ42 significantly (*p* = 0.015) decreased as EMF exposure time increased. (**F**) Aβ40 adjusted by %LDH or CTG. When adjusted by 1-%LDH or CTG, reduction of Aβ40 was not significant (*p* = 0.160 or 0.127, respectively) (**G**) Aβ42 adjusted by %LDH or CTG. When adjusted by 1-%LDH or CTG, reduction of Aβ42 was not significant (*p* = 0.076 or 0.202, respectively).

As per the timeline shown in Fig. 2A, we also examined if REMFS effects on cell viability (measured by the CellTiter Glo (CTG) assay) and toxicity (LDH) and on secretion of Aβ40 and Aβ42 peptides depended upon length of individual exposure sessions. We treated DIV 7 cultures at 64 MHz with a SAR of 0.4 W/kg, for 1 or 2 h, for 14 days, with exposures at 64 MHz and 100 MHz (100 MHz data not shown). CTG data shows no significant change in cell viability dependent on exposure time, while LDH was not significantly elevated (Fig. 2B,C). When Aβ40 and Aβ42 were assayed (Fig. 2D–E), we found that both levels were significantly reduced by REMFS treatment in a time dose-dependent fashion This relationship was not significant when adjusting by either 1-% LDH or CTG as an approximation of overall culture health (Fig. 2F,G). Visual examination of the plots suggested a possible diminishing returns trend, wherein dosage in excess of 1 h or higher than 64 MHz resulted in less optimal results. However, insufficient data points were generated to explicitly test non-linear models.

### REMFS treatments after 7 days of differentiation did not alter sAPPα levels

**Figure 3:**
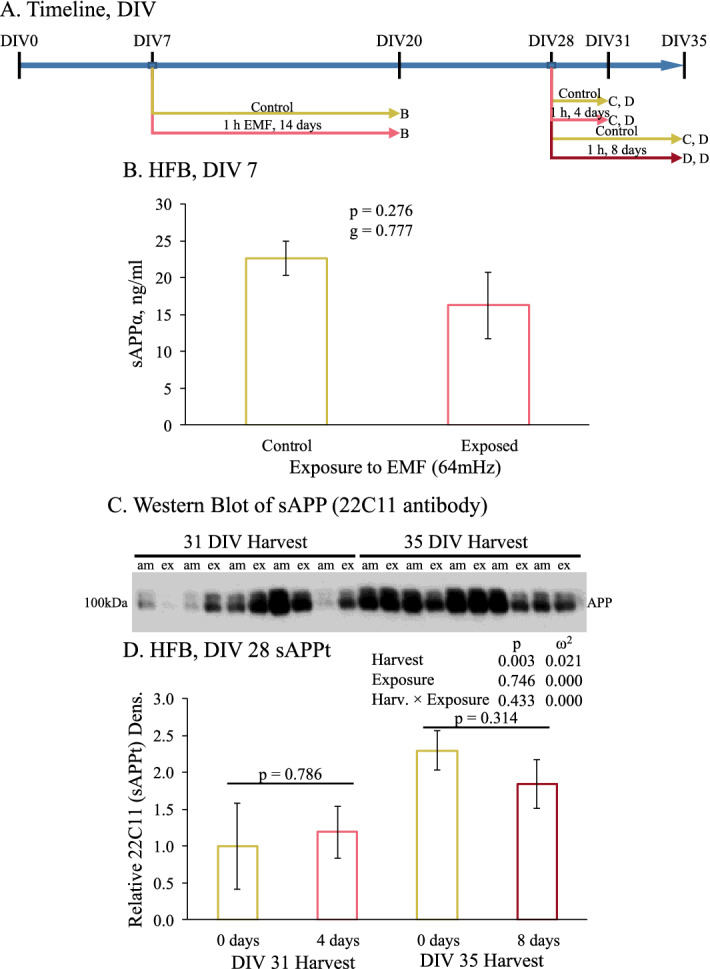
Effect of REMFS on levels of sAPPα and sAPP in human brain cultures at DIV7 and DIV28. Cultures were grown for 7 or 28 days and then exposed as described in the text for 14 (DIV7) or 4 or 8 days (DIV28), as described in the text. CM was assayed for levels of sAPP or sAPPα. sAPPα was measured by ELISA. Total sAPP was measured by semi-quantitative western blotting. (**A**) Schedule of cell growth and treatments for data presented in figure. (**B**) sAPPα was measured by ELISA of CM from PHB-DIV7 exposed to REMF for 14 day as described in the text. REMFS does not cause a significant change in levels of sAPPα. (**C**) Total sAPP was measured by semiquantitative western blotting of CM from PHB-DIV28 exposed to REMFS at 64 MHz with SAR of 0.9 W/kg for 4 or 8 days, as described in the text. (**D**) Analysis of blot densitometry revealed that, while the interval between 28 + 4 and 28 + 8 days significantly increased sAPP, there was no effect of REMFS treatment.

PHB cultures at DIV7 were treated with 1-h daily REMFS at 64 MHz with a SAR of 0.4 W/kg for 14 days (Fig. 3A). On the 14th day of treatment, all conditioned media above the cells was replaced by fresh medium, and total soluble APPα levels were analyzed using ELISA. At exposure day 14, no significant changes in sAPPα levels were observed for the REMFS-treated culture (Fig. 3B).

### REMFS treatments after 28 days of differentiation alter did not alter total APP levels

PHB cultures at DIV 28 cultures were treated with 64 MHz with a SAR of 0.9 W/kg for 4 or 8 days but found no REMF treatment effect on total sAPP levels. As cultures aged overall sAPP increased, regardless of REMFS treatment (Fig. 3C,D).

**Figure 4:**
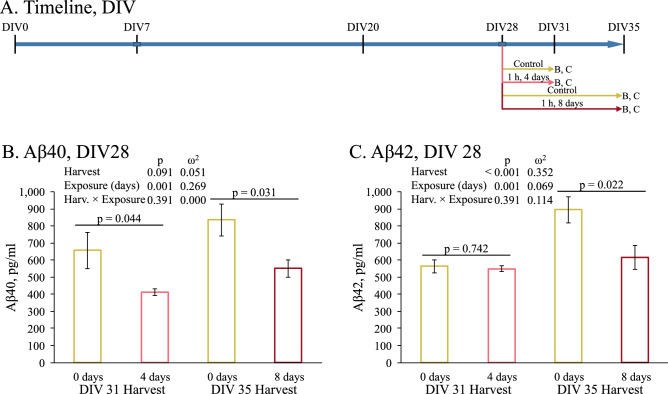
Effect of REMFS on levels of Aβ40 and Aβ42 in human brain cultures at DIV28. Cultures were exposed to REMFS as described in the text (**A**) Schedule of cell growth and treatments for data presented in figure. (**B**) Both culture age (28 + 4 vs 28 + 8 days) and EMF treatment produced significant differences in Aβ40 levels. However, the effect of REMFS treatment was the same regardless of culture age. (**C)** Both culture age (28 + 4 vs 28 + 8 days) and REMFS treatment produced significant differences in Aβ42 levels. However, the effect of REMFS treatment significantly differed by culture age. Reduction only appeared in the oldest cultured cells.

PHB cultures were allowed to grow for 28 days then exposed daily to REMFS at 64 MHz with a SAR of 0.9 W/kg for 1 hour (Fig. 4A). Notably, EMF exposure achieved a significant decrease in the Aβ40 levels (Fig. 4B). This difference was primarily due to length of treatment, as shown by 2-way glm that compared cell harvest at DIV 31 or 35 vs length of REMF exposure (0, 4, or 8 days). While longer additional growth resulted in greater overall Aβ40 (“Day” *p* = 0.048) and REMF exposure reduced Aβ40 levels on each day (*p* = 0.002), the extent of reduction was approximately the same regardless of exposure length (*p* = 0.799 for interaction). Aβ42 levels after 4 and 8 days of REMFS treatments had a different pattern, where culture age (Days) and REMF exposure significantly (*p* = 0.020) interacted. At day four we did not find any difference in Aβ42 levels between the REMFS exposed and control (Naïve) cultures (Fig. 4C). After 8 days of treatment the REMFS cultures showed a significant reduction of the Aβ42 levels compared to the ambient control culture (*p* = 0.022).

## Discussion

The current study is the first, to our knowledge, to show a potentially safe and effective strategy to decrease potentially toxic Aβ levels in primary human brain cultures through application of REMFS. Ultimately, our results revealed that REMFS at 64 MHz with a SAR of 0.4 W/K for 1 hour could reduce levels of secreted Aβ peptides. This minimal energy has valuable clinical implications for the treatment of Alzheimer’s patients, since higher energy levels would induce thermal injuries as well as other potential adverse effects^77^ (Table 1). Importantly, we also found that these treatments did not cause cellular toxicity in PHB cultures, as was noted through analysis of LDH levels.

Separate REMFS schedules were carefully studied to determine the degree of reduction of Aβ40 and Aβ42 levels in PHB cultures, and the treatment chamber and control cultures were maintained in an incubator at all times so as to prevent changes in temperature or environmental electromagnetic frequencies that could potentially alter outcomes. Results in CM samples revealed a 46% reduction of Aβ40 levels when cultures were subjected to REMFS at 64 MHz with a SAR of 0.6 W/kg daily for 1 hour for 14 days and a corresponding 36% reduction in Aβ42. Additional modifiable variables, such as exposure time and frequency were also considered, and the impact of these different EMF settings was studied relative to the reduction in Aβ40 and Aβ42 peptides levels.

While there are differences between mouse models and human tissues, it bears noting that REMFS studies with SAR of 0.25–1.05 W/kg (similar to our study with SAR values of 0.4–0.9 W/kg) reported decreased Aβ levels in older AD mouse models^27–29^. Therefore, we treated PHB cultures differentiated for 28 days to determine if REMFS also reduced Aβ levels in cells near the end of primary culture survival on the dish^78,79^. Results revealed REMFS at 64 MHz with SAR of 0.9 W/kg daily for 1 h after 4 and 8 days produced a significant reduction of Aβ40 levels in the media cultures. Interestingly, a SAR of 0.4 W/kg produced similar results, although a significant reduction of the Aβ42 levels was only noted at day 8. Nevertheless, an overall shorter treatment duration also reduced Aβ levels (4 or 8 vs. 14 or 21 days). This is an advance from our prior results following 21 days of exposure, leading us to believe that through additional fine tuning of REMFS settings in future, the desired biological effects of REMFS may ultimately be achieved after only a few treatments. Conveniently, effects on Aβ deposition could be measured early through analysis of several AD biomarkers, such as amyloid positronic emission tomography (PET), cerebrospinal fluid (CSF)-Aβ (42) and CSF tau levels in patients^80^. Our SAR calculations made specific assumptions (see Methods) regarding density and conductivity. These may not perfectly reflect the differences between brain in living patients vs. monolayer cell culture. Nevertheless, our work gives proof of concept that can be further refined by translational experiments.

Interestingly, we also found that REMFS did not cause a significant change in levels of the sAPPα or total sAPP in PHB cultures. Because sAPPβ (not assayed here) is a unique product of amyloidogenic processing, and sAPPα is a unique product of anabolic APP processing, our findings may suggest several interesting and testable hypotheses for the REMFS-mediated lowering of Aβ levels. One such hypothesis suggests the lowering of Aβ levels may be due to activation of Aβ degradation pathways^34,36,62–64,81–83^ rather than a reduction of APP expression. Other potential pathways could involve inflammation and microglial activation. However, the literature is not yet clear. Exposure of primary neurogenic cell cultures resulted in reduced microglial phagocytic ability and reduced axon lengths and branchpoints^84^. On the other hand, 8-month whole-body exposure of aged mice to REMF had no effects on oxidative stress, apoptosis, or microglia markers versus un-exposed animals^85^. However, the levels of sAPPα and sAPPβ were not compared side-by-side to determine relative changes. Given that sAPPα and total sAPP levels in our conditioned medium did not show a significant reduction, we expected the changes in this product would have pointed to the redirection of APP processing pathway selection^86^.

One such Aβ degradation pathway involves heat shock factor 1 (HSF1); however, it is speculative at this time. Some studies suggest that REMFS decreases Aβ production^25,44^. Given that decreased clearance of Aβ^87^ and loss of proteostasis due to age-related attenuation of the HSF1 pathway are early molecular events in LOAD^88–91^, one could expect that upregulation of the HSF1 pathway in senescent cells^34^ would increase levels of HSPs and chaperones that transport Aβ40 and Aβ42 to the proteasome for degradation, thereby reducing Aβ levels and potentially preventing or ameliorating AD^92–95^.

Overexpression of HSF1 significantly reduced Aβ levels in AD mouse models^96^. Additional evidence suggests REMFS may reactivate the HSF1 pathway and recover its proteostasis activity in senescent cells^34^, and organisms such as old AD mouse models^28^. REMFS may induce these effects by causing structural changes of heat-induced long non-coding RNA 1 (HSR1)^83^, which ultimately binds and activates HSF1 thereby increasing the expression of chaperones such as HSP70 that promote Aβ degradation^97^.

Interestingly, when human peripheral blood mononuclear cells derived from AD patients were exposed to pulsed EMF, upregulation of microRNA (miR)-107 and reduced levels of BACE1 mRNA were observed^44^. Also, whole-body exposure of rats to EMF upregulated miR-107 in brain tissues^46^. MiR-107has been shown to downregulate BACE1 translation^98,99^.

As mentioned, other electromagnetic stimulation methods may also be useful versus AD, but they would operate through different pathways than REMF. TMS, for example, induces an electric current that depolarizes neurons and trigger action potentials using a field strength of about 1 tesla (T), it allows stimulating the brain areas located up to 2 cm from its surface^100^. REMFS may not depolarize neurons. It radiates low energy coupled electromagnetic fields with non-thermal effects at a frequency of 64 MHz that activate intracellular biomolecules; it allows to stimulate the brain areas located up to 13.49 cm from its surface^73^. Secondly, TMS treatment significantly decreased levels of APP in AD mice treated with TMS: 67.1 ± 10.0% relative to non-treated mice, *p* < 0.05^101^. There were no significant changes in the APP levels in our REMFS experiments, also suggesting a different mechanism of decreasing Aβ aggregates; however, models are different.

Another type of non-pharmacological intervention is Deep Brain Stimulation (DBS), which also uses electrical currents for stimulation. DBS is a well-established neurosurgical technique used to treat neurological disorders such as Parkinson’s disease^102,103^. A recent study on the effects of the electrical stimulation on neural precursor cells found that there was a twofold increase in the neural stem cell pool and increase in neurogenesis under direct current stimulation of 250 mV/mm, these findings suggest a regenerative strategy to neural repair^105^. Finally, REMFS approach might complement within a broad context of other strategies, such as diazoxide, melatonin, resveratrol, and nanocurcumin, tested in different models^105–107^.

In short, precise mechanisms by which REMFS lowers Aβ levels in the PHB culture are unclear by any measure. Also, we recognize the limitation of our present work on several fronts. First, the number of samples is low (n = 3–4). This is partly due to the small area of the TCM chamber, which has limited room to accommodate several multi-well plates at the same time. Since we aimed at performing every experiment under identical conditions, we avoided doing experiments in batches with time intervals and then pooling samples. We were, thus, constrained regarding testing different conditions such as power, frequency and SAR settings. Therefore, future work with a redesigned large TCM chamber is needed. Second, we used primary human brain cultures throughout our experiments. This is an important innovation as primary culture derived from human fetal brain tissue is much closer to human AD than humanized transgenic animal models. We must note that PHB has become a regulatory challenge. Third, we could not measure several other proteins, such as HSTF1 (which requires nuclear extracts), as discussed in the text, due to the low amount of proteins derived from each well of the dish containing primary neurons. Nevertheless, the preliminary results from this experiment would encourage other investigators of the field to move this idea further. If REMFS produces the effects we observed on Aβ primarily through increased protein turnover, our findings could have implications for the treatment of other protein-associated neurodegenerative disorders associated with aberrant protein accumulations^108^. On the other hand, there is also a possibility of an AD-specific mechanism^44,109^. Finally, given the multitarget nature of REMFS, a synergistic modulation of both pathways is possible, as evidenced in previous studies.

## Methods

### Culture of primary human brain (PHB) cells

The protocol was approved by the Indiana University School of Medicine Institutional Review Board (IRB) and complied with state and federal regulations. Primary cultures of mixed human fetal brain cells were prepared from the brain parenchyma of aborted fetuses (80–110 days gestational age), as described previously^76^. The tissues were obtained from the Laboratory of Developmental Biology, University of Washington, Seattle, WA, after shipping overnight in chilled Hibernate-E medium (Invitrogen) supplemented with B27 (Invitrogen), GlutaMAX (Invitrogen), and antibiotic/antimycotic solution (Cellgro). All samples were collected under the supervision of the IRB of the University of Washington, which collected and keeps on file all appropriate informed consent. The meninges and blood vessels were stripped off; the brain tissue was washed in minimum essential medium and enzymatically dissociated by incubation in 0.05% Trypsin- 0.53 mM EDTA solution at 37 °C in a shaking water bath set to 150RPM. Tissue was subsequently mechanically dissociated by trituration through a siliconized (Sigma-Cote; Sigma-Aldrich, St Louis, MO), fire-polished Pasteur pipette.

Cells were then centrifuged at 800×*g* for 10 min, resuspended and seeded at an initial density of 2.2 × 10^5^ cells/cm^2^ in Neurobasal (plus GlutaMAX, B27, antibiotic cocktail, normocin, bFGF) and allowed to attach overnight in poly-d-lysine (PDL) coated 24-well tissue culture plates. The following day, media and non-cellular debris were aspirated from the plate and media replaced with Neurobasal medium (Invitrogen), supplemented with 1× B27, 0.5 mM GlutaMAX, 5 ng/ml basic FGF (Invitrogen), and antibiotic/antimycotic mixture. Half-media changes were performed every 3rd day of culture. PHB cultures have been shown previously to comprise approximately 60 to 70% neurons with 30 to 40% mixed glial cells and have been established as a physiological model for growth of neurons and supporting cells^79^. In these cultures there is initially rapid neuronal growth, followed by a plateau/small decline before marked decline coupled with gliosis at 70 to 84 days in vitro^76^. Sample sizes were chosen to provide adequate power based on our prior work with PHB cultures^110^. Post-treatment as indicated, conditioned media was collected by pipette and stored, cells were washed with 1× PBS and lysed using 100 µL of Mammalian protein extraction reagent (M-Per, Life technologies) containing one tablet of protease inhibitor cocktail (Roche). The cell lysate was centrifuged for 10 mins at 30,000*g* and the supernatant was collected and used for further assays.

### Electromagnetic field exposures and treatment conditions

**Figure 5:**
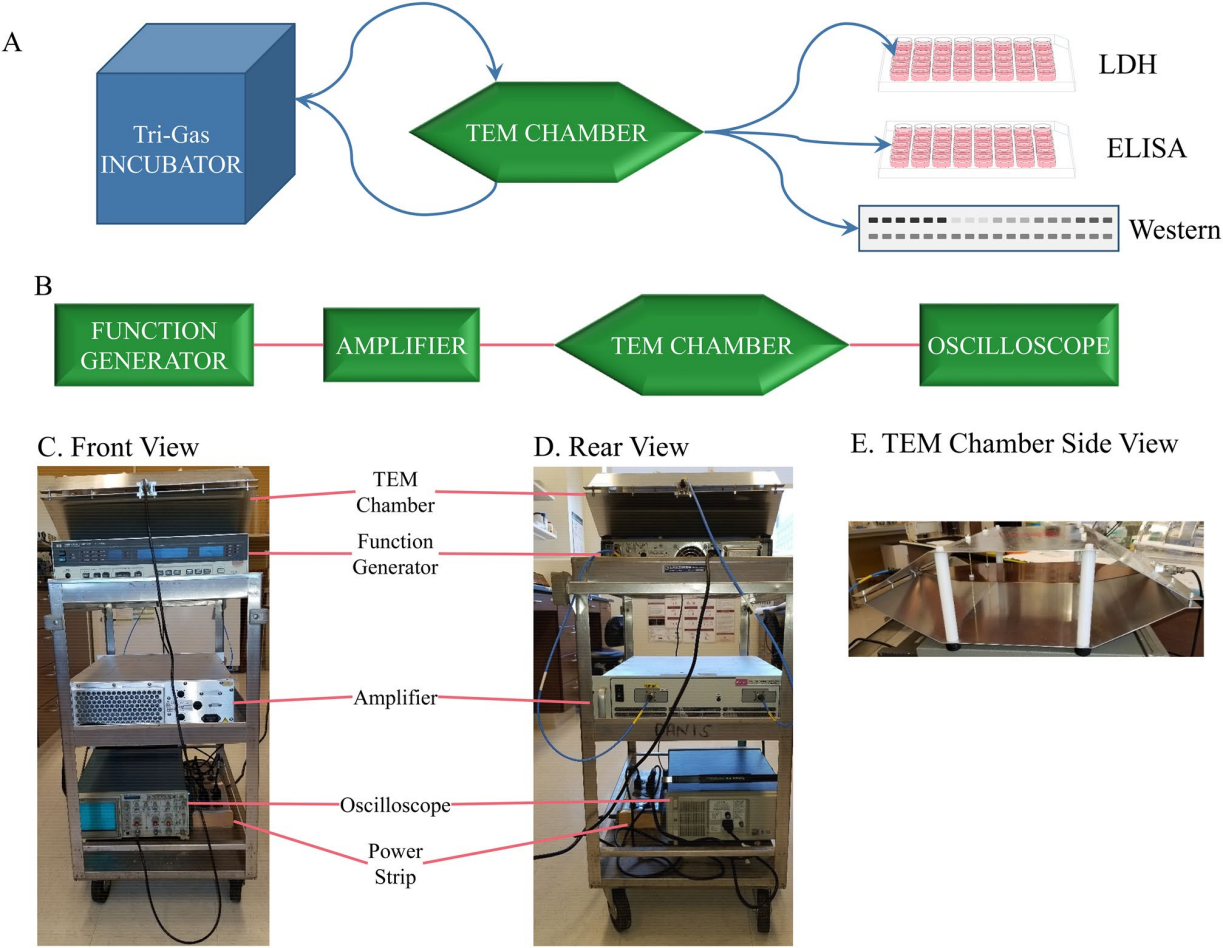
REMFS experiment workflow and apparatus. (**A**) Workflow began with culturing cells in tri-gas incubator, alternating with treatments in TEM chamber. After final treatments, cells were processed and extracts used for analysis by LDH (cell death), ELISA (Aβ) and western blotting (APP). (**B**) Schematic diagram illustrating the source of the electromagnetic fields (function generator). The signal is then sent through an amplifier, then through the TEM chamber. Signal is monitored through the TEM chamber with an oscilloscope. (**C**) Front view photograph of a compact and convenient equipment system. The TEM chamber, being very light, rests upon the function generator. The next lower shelf holds the amplifier. The oscilloscope rests on the bottom shelf. Appropriate cables link each component. Power is supplied by a permanently affixed power strip on the bottom cart shelf. (**D**) Rear view of the compact cart setup. (**E**) Side view of the TEM chamber, showing shelf running across middle.

Electromagnetic field exposures were carried out using a vertically-mounted IFI TEM Cell (Transversal Electromagnetic Cell, model CC110-SPEC, DC to 1000 MHz, Test Equipment Corporation, Mountain View, CA, IFI Ronkonkoma NY). This chamber is an expanded coaxial transmission line operating in the TEM mode, consisting of a main rectangular waveguide that contains a flat-metal-strip center conductor located in the middle between the top and bottom walls. The wall and center conductor are tapered at both ends to provide 50-Ω impedance along the entire length of the chamber. One port was connected to the RF source (HP 8656B/57A/57B synthesized signal generator) via coaxial cable and the other end to a matched load impedance of 50-ohms (provided by an oscilloscope), which is the characteristic impedance to mimic free space or plane wave irradiation. The complete array was mounted on a compact and portable cart (Fig. 5). The wave impedance throughout the chamber is the 377-ohms intrinsic impedance of free space^34,111^. We used the constants of 1030 kg/m^3^ for density of culture medium and 1.15 S/m for conductivity (derived from brain tissues) for our calculation of SAR values^34,73^.

**Figure 6:**
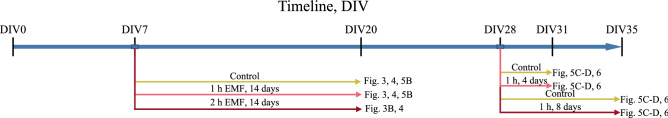
Schedule of treatments of tissue cultures by REMFS. Our study used different aged cultures (measured by days in vitro, DIV), different exposure times per exposure, and different days of repeated exposure. Figure keys each combination to specific results figures (Fig. 3–4). DIV0 is day tissues were triturated and initially seeded.

PHB cultures were subjected to REMFS (Fig. 6) at 64 MHz and 100 MHz, with different times (1 or 2 h) and exposure schedules (daily for 4, 8, or 14 days). We used power levels of 0.125, 0.5, and 1 Watts for our experiments with SAR of 0.4, 0.6, and 0.9, respectively. Levels of Aβ were measured in conditioned medium (CM) samples after 4, 8, 14, and 21 days of exposure in treated and control cultures. Temperature and SAR exposures were derived from computer simulations in our recent paper^73^. The 14- and 21-day exposures were performed on PHB cultures beginning DIV 7, while 4- and 8-day exposures were on HFB cultures beginning DIV28.

### ELISA of Aβ40 and Aβ42 peptides

Levels of Aβ40 and Aβ42 were measured using specific ELISA kits obtained from IBL (catalogue #s 27713 and 27711, respectively), and the assay was performed as per the manufacturer’s protocol. Briefly, an equal volume (50 μl) of conditioned medium was added onto the well, which was pre-coated with monoclonal anti-human Aβ (35–40) antibody (clone 1A10) for Aβ40 or polyclonal rabbit IgG to Aβ (38–42) for Aβ42 and incubated overnight. HRP-conjugated monoclonal anti-human Aβ (11–28, clone 12B2) or mouse polyclonal anti-Aβ (11–28) were used as detection antibodies for Aβ40 or Aβ42, respectively^78,112,113^. The assays can detect as low as 5 pg/ml of Aβ40 or 4 pg/ml of Aβ42 in a typical culture sample with cross-type reactivities (Aβ 40 vs. 42) of < 0.2%. Absolute Aβ values (pg/ml of CM) were measured and corrected for well-to-well variations in cell number by either normalizing to total protein as measured by BCA. We read colorimetric signals of all ELISAs at 450 nm on a microplate reader (BioRad, Model 550).

### Determination of cellular toxicity and viability

LDH enzyme is a cytosolic component of the glycolytic pathway, and leakage from the cytoplasm into the cell culture medium is an indication of membrane permeability, which results from cellular toxicity. The CTG assay measures ATP presence by luminescent reaction. ATP is taken as an indicator of cell viability. For LDH, after 14 days of REMFS treatments, CM samples (50 µl) were collected from treated and control cultures. To determine cellular toxicity and/or membrane damage, LDH was measured in the CM as well as cell lysate samples using the Tox-7 kit (Sigma-Aldrich, St. Louis, MO). Leakage of cytosolic LDH enzyme from the membrane would indicate toxicity and membrane damage^113^. For CTG, after 14 days of REMFS treatments, cells from the same wells used for LDH and other measurements of conditioned media were harvested and lysed in M-PER buffer, clarified, and lysates used for CTG (Promega G7570) assay and measured with Glomax luminometer.

### Statistical analyses

Data are presented as means ± SEM. We performed hypothesis testing with generalized linear models (glm) followed by Dunnett’s test or (Šidak-protected) Student’s *t* test, as appropriate and considered *p* ≤ 0.05 to indicate statistical significance. In addition, we calculated Hedge’s *g* or ω^2^ as appropriate for standardized effect sizes^114^. Further testing by second-order polynomial models was performed if the second-order Akaike information criterion (AICc) for a model with an orthogonal polynomial.

## Supplementary information

Supplementary Information.

## Data Availability

Data is available from the corresponding author on request.

## Abbreviations

AD: Alzheimer’s disease
APP: Aβ precursor protein
Aβ: Amyloid-β
BACE1: Beta-site APP cleaving enzyme 1 or β-Secretase
CM: Conditioned medium
CSF: Cerebrospinal fluid
EOAD: Early-onset
FDA: Food and Drug Administration
PHB: Primary human brain
HSF1: Heat shock factor 1
IEC: International Electrotechnical Commission
IRB: Institutional Review Board
LDH: Lactate dehydrogenase
LOAD: Late Onset Alzheimer’s Disease
MRI: Magnetic resonance imaging
MSBE: Minimum SAR with biological effect
NBS: Noninvasive brain stimulation
PET: Positronic emission tomography
REMFS: Repeated electromagnetic field stimulation
sAPPα: Secreted Aβ precursor protein-α
SAR: Specific absorption rate
TEM: Transversal electromagnetic
TMS: Transcranial magnetic stimulation
τ: Microtubule-associated protein tau
