# Supplementary material for: Repeated electromagnetic field stimulation lowers amyloid-β peptide levels in primary human mixed brain tissue cultures
Source: Sci Rep. 2021 Jan 12;11:621. doi: 10.1038/s41598-020-77808-2 (PMC7804462; doi:10.1038/s41598-020-77808-2)
Supplement: Supplementary file 1 — Supplementary Information. [file 41598_2020_77808_MOESM1_ESM.pdf]

REPEATED ELECTROMAGNETIC FIELD STIMULATION LOWERS AMYLOID-BETA PEPTIDE LEVELS IN PRIMARY HUMAN NEURON-RICH BRAIN TISSUE CULTURES

Felipe P. Perez,[2] Bryan Maloney,[1] Nipun Chopra,[1] Jorge J. Morisaki,[3] and Debomoy K. Lahiri[1]

[1]Department of Psychiatry, Institute of Psychiatric Research, Neuroscience Research Center, Indiana University School of Medicine, Indianapolis, IN, USA.

[2]Depatment of Medicine, Division of General Internal Medicine and Geriatrics, Aging for Center Research, Indiana University School of Medicine, Indianapolis, IN, USA.

[3]Department of Bioengineering, University of Illinois at Chicago, Chicago, IL, USA.

Corresponding author

Dr. D. K Lahiri

Neuroscience Research Center

320 W. 15th St., Indianapolis IN 46201, USA

Tel: (317) 274-2706

E-mails: dlahiri@iupui.edu

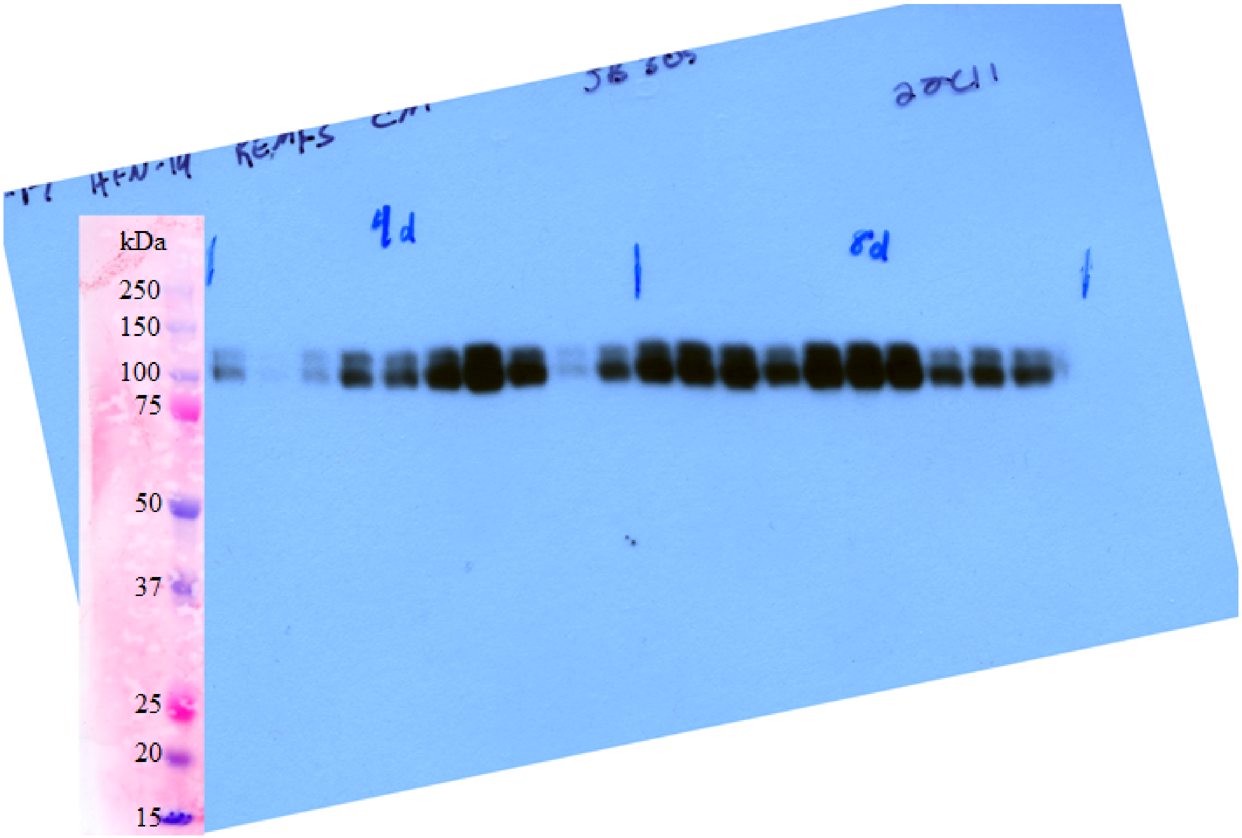

Fig. S1. Total sAPP was measured by semiquantitative western blotting of CM from PHB-DIV28 exposed to REMFS at 64 MHz with SAR of 0.9 W/Kg for 4 or 8 days, as described in the main text. Un-edited scan of X-ray film with superimposed protein MW standard (BioRad 1610374) scanned from Ponceau-S stain of blot before antibody probing.
